# Targeted ablation of p38α MAPK suppresses denervation-induced muscle atrophy

**DOI:** 10.1038/s41598-018-26632-w

**Published:** 2018-06-13

**Authors:** Kazuki Yuasa, Kazumasa Okubo, Masaki Yoda, Kinya Otsu, Yasuyuki Ishii, Masaya Nakamura, Yoshiki Itoh, Keisuke Horiuchi

**Affiliations:** 1 Pharmacological R&D Section, Pharmaceutical Research Department, Sato Pharmaceutical Co., Ltd., 6-8-5 Higashi-ohi, Shinagawa, Tokyo, 140-0011 Japan; 2 Department of Orthopedic Surgery, Keio University School of Medicine, 35 Shinanomachi, Shinjuku, Tokyo, 160-8582 Japan; 3 Laboratory of Cell and Tissue Biology, Keio University School of Medicine, 35 Shinanomachi, Shinjuku, Tokyo, 160-8582 Japan; 4 The School of Cardiovascular Medicine and Sciences, King’s College London, Strand, London, WC2R 2LS UK; 5 Drug Discovery Research Department, Sato Pharmaceutical Co., Ltd., 6-8-5 Higashi-ohi, Shinagawa, Tokyo, 140-0011 Japan; 6 Department of Orthopedic Surgery, National Defense Medical College, 3-2 Namiki, Tokorozawa, Saitama, 359-8513 Japan

## Abstract

The loss of skeletal muscle mass is a major cause of falls and fractures in the elderly, leading to compromised independence and a decrease in the quality of life. However, only a few therapeutic interventions leading to marginal clinical benefits in patients with this condition are currently available. Therefore, the demand to further understand the pathology of muscle atrophy and establish a treatment modality for patients with muscle atrophy is significant. p38α mitogen-activated protein kinase (p38α MAPK) is a ubiquitous signaling molecule that is implicated in various cellular functions, including cell proliferation, differentiation, and senescence. In the present study, we generated a mutant line in which p38α MAPK is specifically abrogated in muscle tissues. Compared with the control mice, these mutant mice are significantly resistant to denervation-induced muscle atrophy, suggesting that p38α MAPK positively regulates muscle atrophy. We also identified CAMK2B as a potential downstream target of p38α MAPK and found that the pharmacological inhibition of CAMK2B activity suppresses denervation-induced muscle atrophy. Altogether, our findings identify p38α MAPK as a novel regulator of muscle atrophy and suggest that the suppression of intracellular signaling mediated by p38α MAPK serves as a potential target for the treatment of muscle atrophy.

## Introduction

The loss of muscle mass (i.e., muscle atrophy) is among the most common musculoskeletal disorders. Muscle atrophy is caused by various conditions, including aging, disuse (such as cast immobilization and prolonged bed rest), systemic inflammation, endocrine disorders, cachexia, and hyponutrition. Aging-related muscle atrophy (i.e., sarcopenia) severely compromises the ability of daily living, hampers the quality of life, and, thus, is an increasingly important issue, particularly in aging societies^1,2^. However, other than physical exercise and intake of nutrition, only limited treatment options are available for this condition, and these treatments yield marginal clinical benefits^3,4^. Therefore, understanding the mechanism underlying muscle atrophy and developing effective treatments and prophylaxis for this disorder are crucial.

Recent studies have identified the key signaling molecules and pathways that regulate muscle atrophy and hypertrophy. Among these pathways, signaling pathways mediated by the serine/threonine-specific kinase AKT1 (also known as protein kinase B, PKB) are likely the most important and well investigated^5–8^. Upon activation, AKT1 promotes protein synthesis via the mTOR complex 1 and p70S6K pathway and suppresses protein degradation by inhibiting the activity of the FOXO transcription factor. In contrast, upon deactivation, AKT1 suppresses protein synthesis and induces the expression of the muscle-specific ubiquitin ligases MuRF1 (encoded by *Trim63*) and Atrogin1 (encoded by *Fbxo32*) by activating the FOXO transcription factor, thereby promoting muscle degradation^9,10^. At the cellular level, various endogenous and exogenous stimuli, such as the accumulation of reactive oxygen species and signaling from inflammatory cytokines and growth factors, are intricately involved in the regulation of this signaling pathway.

To address these stimuli, cells are equipped with intracellular mediators that are largely dependent on protein phosphorylation. The mitogen-activated protein kinase (MAPK) family proteins, which are evolutionarily conserved serine/threonine protein kinases, play a central role in this signal transduction pathway^11,12^. In mammals, the three major classes of MAPKs are as follows: the extracellular signal-regulated kinases (ERKs), the c-Jun N-terminal kinases (JNKs), and the p38 MAPKs. The p38 MAPK family members (i.e., p38α, p38β, p38γ, and p38δ MAPKs) function as transducers of cellular stress and various non-stress related stimuli. Accordingly, the p38 MAPK pathway has diverse functions and is implicated in various cellular processes, including senescence, apoptosis, cell-cycle arrest, inflammatory reaction, and tumorigenesis^12^.

p38α MAPK has been shown to function as a positive regulator of satellite cell differentiation^11,13–15^. Mice lacking *Mapk14* specifically in satellite cells exhibit smaller skeletal muscles and an increased satellite cell pool^16^. These mice can respond to muscle injury; however, muscle repair is significantly delayed due to the hampered satellite cell differentiation. Furthermore, the aberrant cell-autonomous activation of p38 MAPKs is associated with the aging of satellite cells^17,18^. The p38 MAPK pathway has also been implicated as a potential catabolic signaling mediator in skeletal muscle^19^. Consistently, the p38 MAPK pathway mediates the TNFα-induced expression of MuRF1 and Atrogin1^20,21^, and the p38α MAPK activity is elevated in skeletal muscles in various murine muscle atrophy models likely due to the accumulation of reactive oxygen species or the stimulation of inflammatory cytokines^22–26^. Although these studies suggest that p38α MAPK is potentially involved in the regulation of muscle atrophy, this hypothesis has not been fully explored *in vivo*.

In the present study, we aimed to elucidate the physiological functions of p38α MAPK in skeletal muscle atrophy. Because p38α MAPK is ubiquitously expressed *in vivo*, we generated a mutant line in which the *Mapk14* allele is specifically excised under the control of a human muscle actin promoter. p38α MAPK in skeletal muscle is activated after denervation, and the mutant mice exhibit resistance to denervation-induced muscle atrophy. Furthermore, we also identified CAMK2B, which is a member of the calcium/calmodulin-dependent protein kinase II (CaMKII) family, as a potential downstream target of p38α MAPK and found that the pharmacological inhibition of CaMKII suppresses denervation-induced muscle atrophy. Altogether, our findings establish p38α MAPK as a positive regulator of muscle atrophy and suggest that the suppression of the intracellular signaling mediated by p38α MAPK serves as a potential therapeutic target for muscle atrophy.

## Results

### Muscle-specific abrogation of p38α MAPK in mice does not lead to overt developmental defects

**Figure 1:**
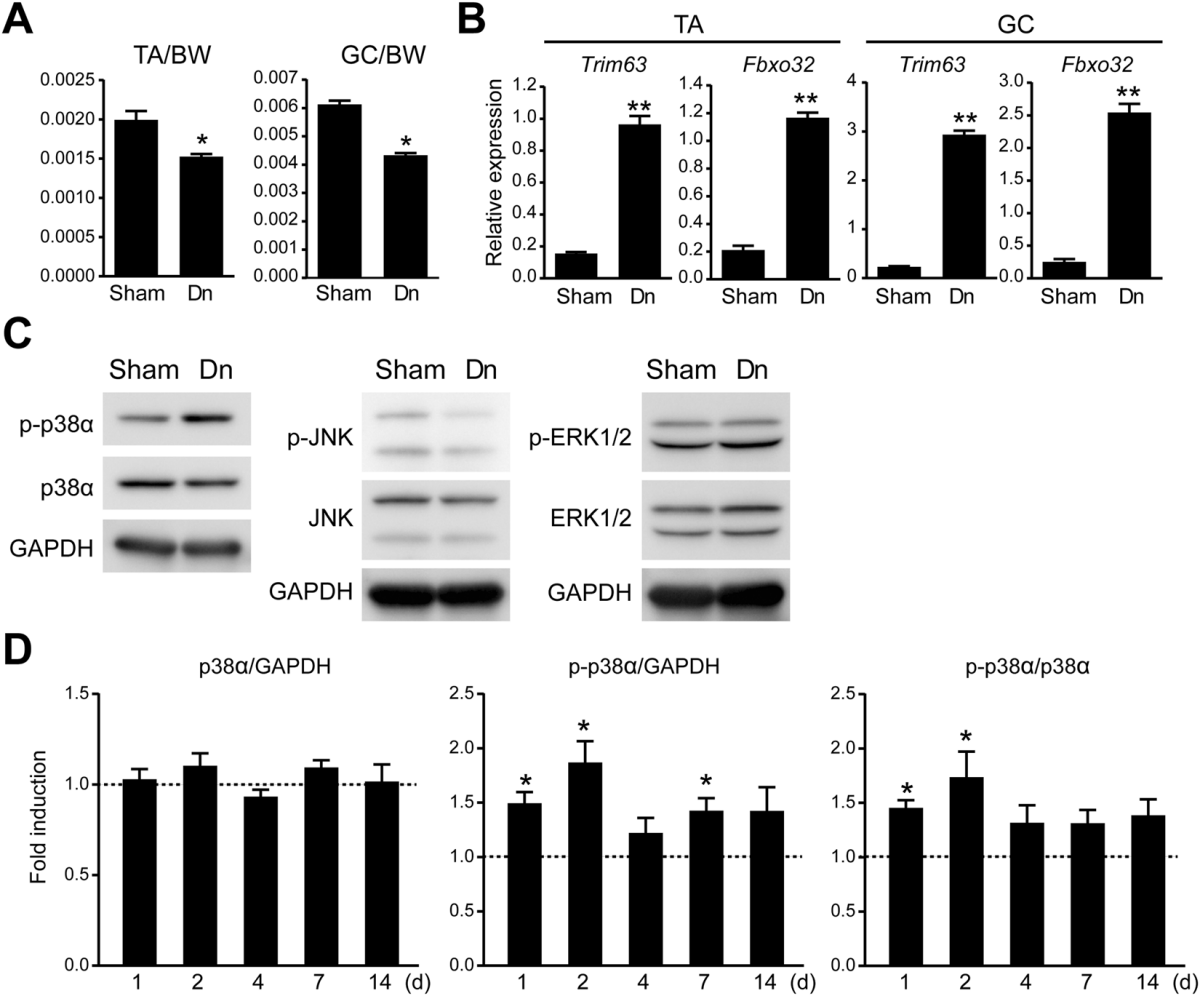
p38α MAPK is activated in skeletal muscle after denervation. **(A**) The ratio of the weight of the TA and GC muscles to the BW. **(B**) Relative expression of *Trim63* and *Fbxo32* transcripts in the TA and GC muscles 4 d after treatment. n = 5 mice per treatment group. **(C**) Expression of phosphorylated- (p-) and total-MAPKs in the TA muscle collected 24 h after treatment. Representative results of 3 independent experiments are shown. **(D**) Quantitative analysis of the expression level of p38α MAPK and phosphorylated- p38α MAPK in the TA muscle 1, 2, 4, 7, and 14 d after denervation. The data are shown as the band intensity ratios of p38α MAPK: GAPDH, phosphorylated- p38α MAPK: GAPDH, and phosphorylated- p38α MAPK: p38α MAPK. The average value of the sham-operated TA specimens at each time point is set to 1. n = 5 mice at each time point. Sham, sham surgery; Dn, denervation. *p < 0.05, **p < 0.005.

To investigate the potential involvement of p38α MAPK in muscle atrophy, we used a sciatic nerve denervation model to induce muscle atrophy in mice. We confirmed that a significant decrease in muscle mass can be achieved after denervation (Fig. 1A). Consistently, the transcripts of the muscle-specific E3 ubiquitin ligases, i.e., *Trim63* (encoding MuRF1) and *Fbxo32* (encoding Atrogin1), that are critically involved in muscle atrophy, were significantly induced in the tibialis anterior (TA) and gastrocnemius (GC) muscles after denervation (Fig. 1B). Next, we examined the effects of denervation on the activity of the MAPKs that are involved in the cellular stress response in skeletal muscle. According to a Western blot analysis, the phosphorylation of p38α MAPK, but not of JNK or ERK1/2, is induced by denervation in the TA muscle, suggesting that p38α MAPK is potentially involved in denervation-induced muscle atrophy (Fig. 1C). Furthermore, we also quantitatively examined the time course changes of the expression levels of p38α MAPK and phosphorylated p38α MAPK and found that the expression level of p38α MAPK remains static and that the phosphorylation of p38α MAPK peaks at around 1–2 days after denervation (Fig. 1D).

**Figure 2:**
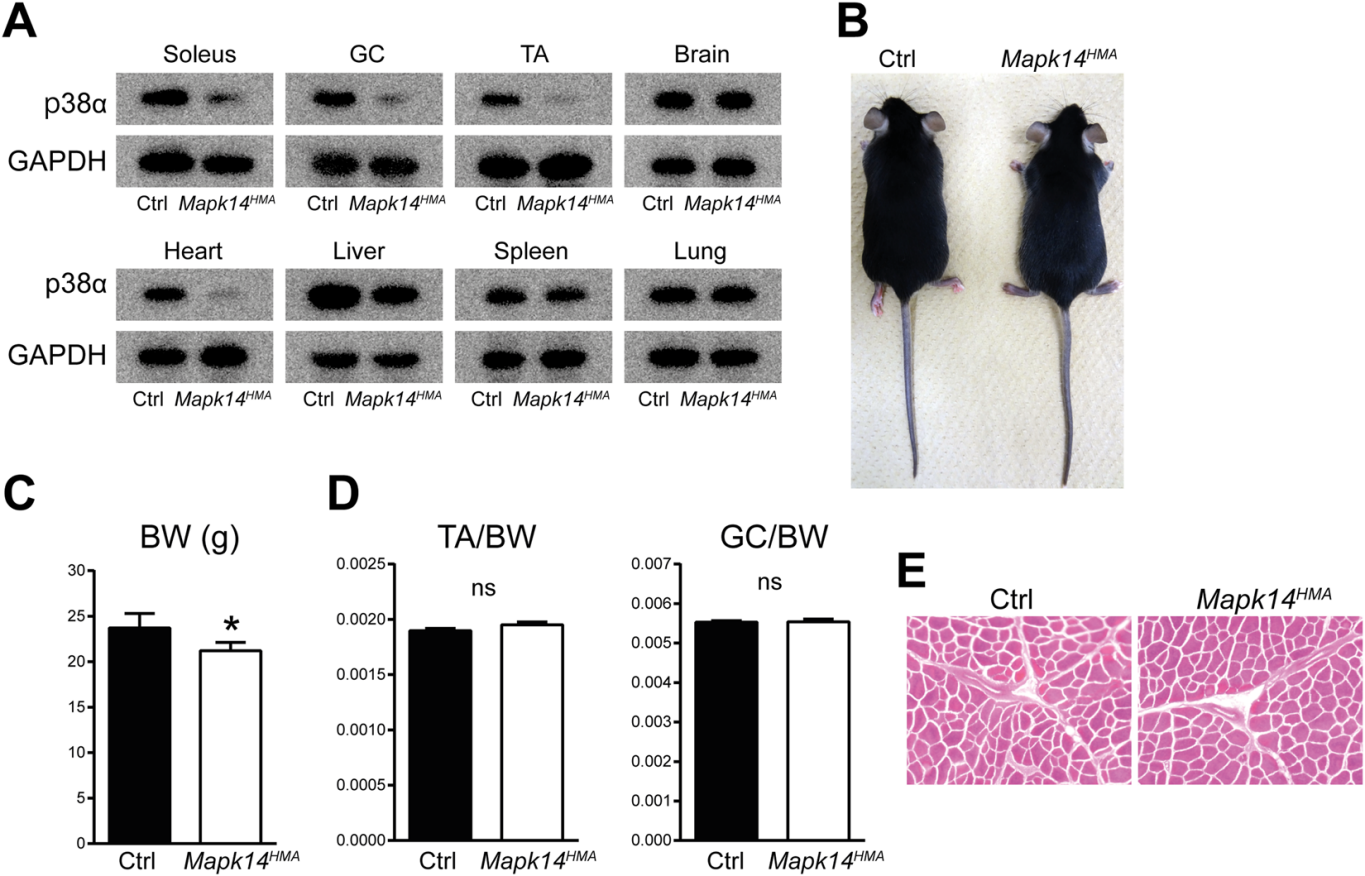
*Mapk14*^*HMA*^ mice exhibit no apparent defects. (**A**) p38α MAPK expression in the soleus, GC, and TA muscles, brain, heart, liver, spleen, and lung collected from 7-week-old Ctrl and *Mapk14*^*HMA*^ mice. (**B**) gross appearance of 7-week-old male Ctrl and *Mapk14*^*HMA*^ mice. (**C,D**) BW (**C**) and the ratio of the TA and GC muscle to the BW (**D**) of 7-week-old Ctrl and *Mapk14*^*HMA*^ mice. n = 6 (Ctrl) and 7 (*Mapk14*^*HMA*^) mice. (**E**) H&E stained sections of the TA muscle collected from the Ctrl and *Mapk14*^*HMA*^ mice. *p < 0.05.

Because p38α MAPK is activated upon muscle denervation, we generated mutant mice in which p38α MAPK is specifically inactivated in skeletal muscles under the control of a human alpha-skeletal actin promoter by mating *Mapk14*^*flox/flox*^ mice^27^ with B6.Cg-Tg (ACTA1-cre) 79Jme/J transgenic mice (*Mapk14*^*HMA*^ mice)^28^ to elucidate the contribution of p38α MAPK to muscle atrophy *in vivo*. The *Mapk14*^*flox/flox*^ mice exhibit no obvious defects and were used as control (Ctrl) animals in the present study. The muscle-specific abrogation of p38α MAPK in the *Mapk14*^*HMA*^ mice was confirmed by a Western blot analysis (Fig. 2A). The *Mapk14*^*HMA*^ mice were fertile and exhibited no apparent defects, suggesting that p38α MAPK in skeletal or cardiac muscles is not essential for developmental or post-natal growth in mice (Fig. 2B and data not shown). However, a slight but statistically significant decrease in body weight (BW) was observed in the *Mapk14*^*HMA*^ mice compared with the Ctrl mice (Fig. 2C). Nevertheless, no marked differences were observed in the muscle (TA or GC muscles) weight to body weight ratio or the histology of the muscle tissues between the *Mapk14*^*HMA*^ mice and the Ctrl mice (Fig. 2D,E).

### *Mapk14*^*HMA*^ mice are more resistant to denervation-induced muscle atrophy than Ctrl mice

**Figure 3:**
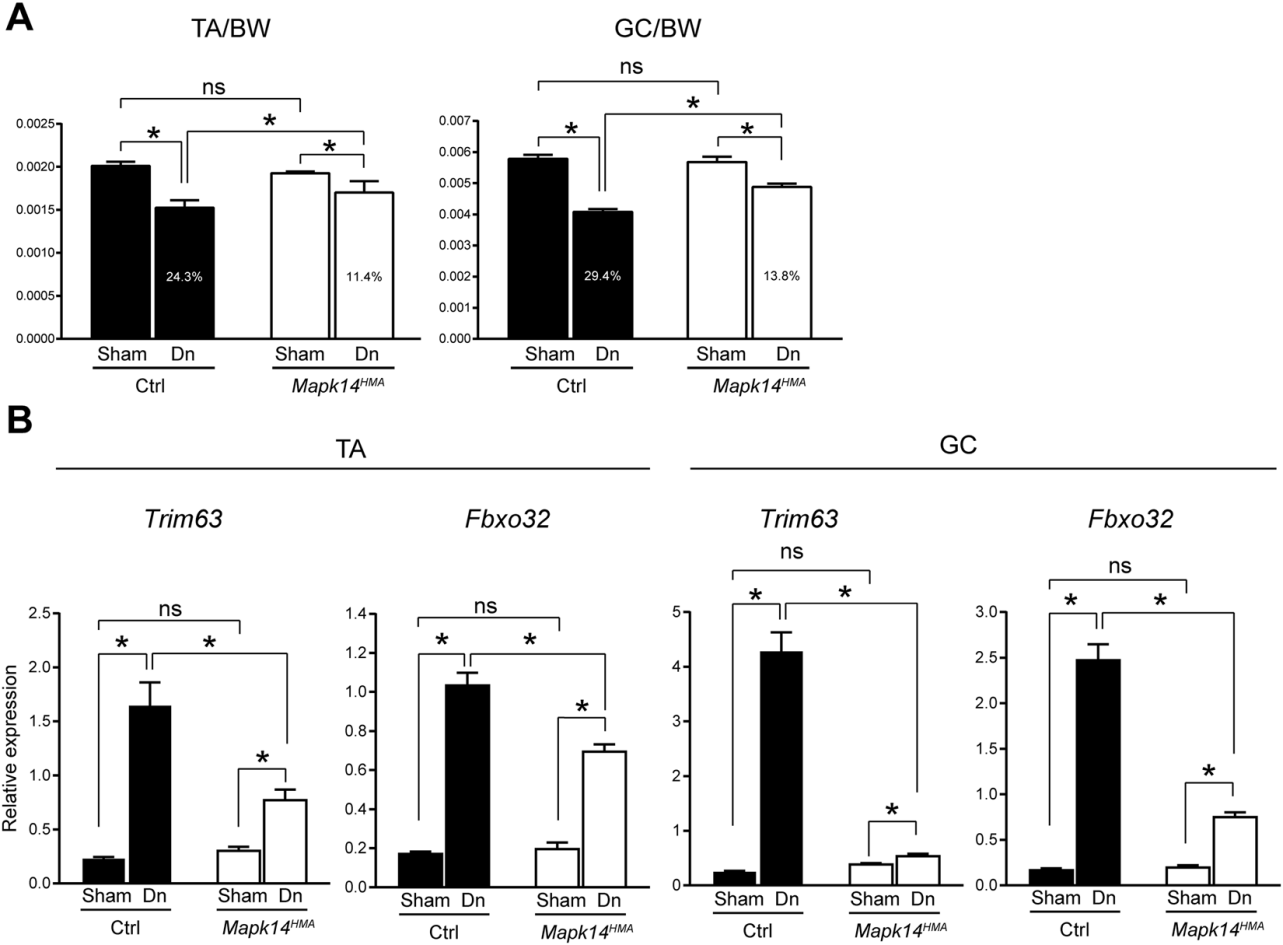
*Mapk14*^*HMA*^ mice are less sensitive to denervation-induced muscle atrophy than Ctrl mice. **(A**) The ratio of the TA and GC muscles to the BW 7 d after treatment. Numbers in the columns indicate the difference (%) between the sham-operated (set to 100%) and denervated specimens. n = 6 per genotype. **(B**) Relative *Trim63* and *Fbxo32* expression in the TA and GC muscles 4 d after treatment. n = 6 (Ctrl) and 7 (*Mapk14*^*HMA*^) mice. Dn, denervation. ns, not significant, *p < 0.05.

The *Mapk14*^*HMA*^ and Ctrl mice were subjected to sciatic nerve denervation as described in the Experimental procedures. The muscle tissues were collected for analysis 4 d (for gene expression analysis) or 7 d (for muscle weight analysis) after the intervention as described in the Methods. No significant difference was observed in the muscle weight to BW ratio in the sham-operated limbs between the *Mapk14*^*HMA*^ and Ctrl mice (Fig. 3A). In contrast, the muscle weight to BW ratio in the denervated limbs was significantly higher in the *Mapk14*^*HMA*^ mice than that in the Ctrl mice, indicating that the effects of denervation on muscle atrophy are attenuated in the *Mapk14*^*HMA*^ mice. Consistently, the MuRF1 and Atrogin1 transcripts were markedly lower in the muscle collected from the *Mapk14*^*HMA*^ mice than those in the muscle from the Ctrl mice (Fig. 3B). Thus, the *Mapk14*^*HMA*^ mice are more resistant to denervation-induced muscle atrophy than Ctrl mice, and p38α MAPK is involved in the regulation of muscle atrophy *in vivo*.

### p38α MAPK regulates muscle atrophy by mediating CAMK2B activity

**Figure 4:**
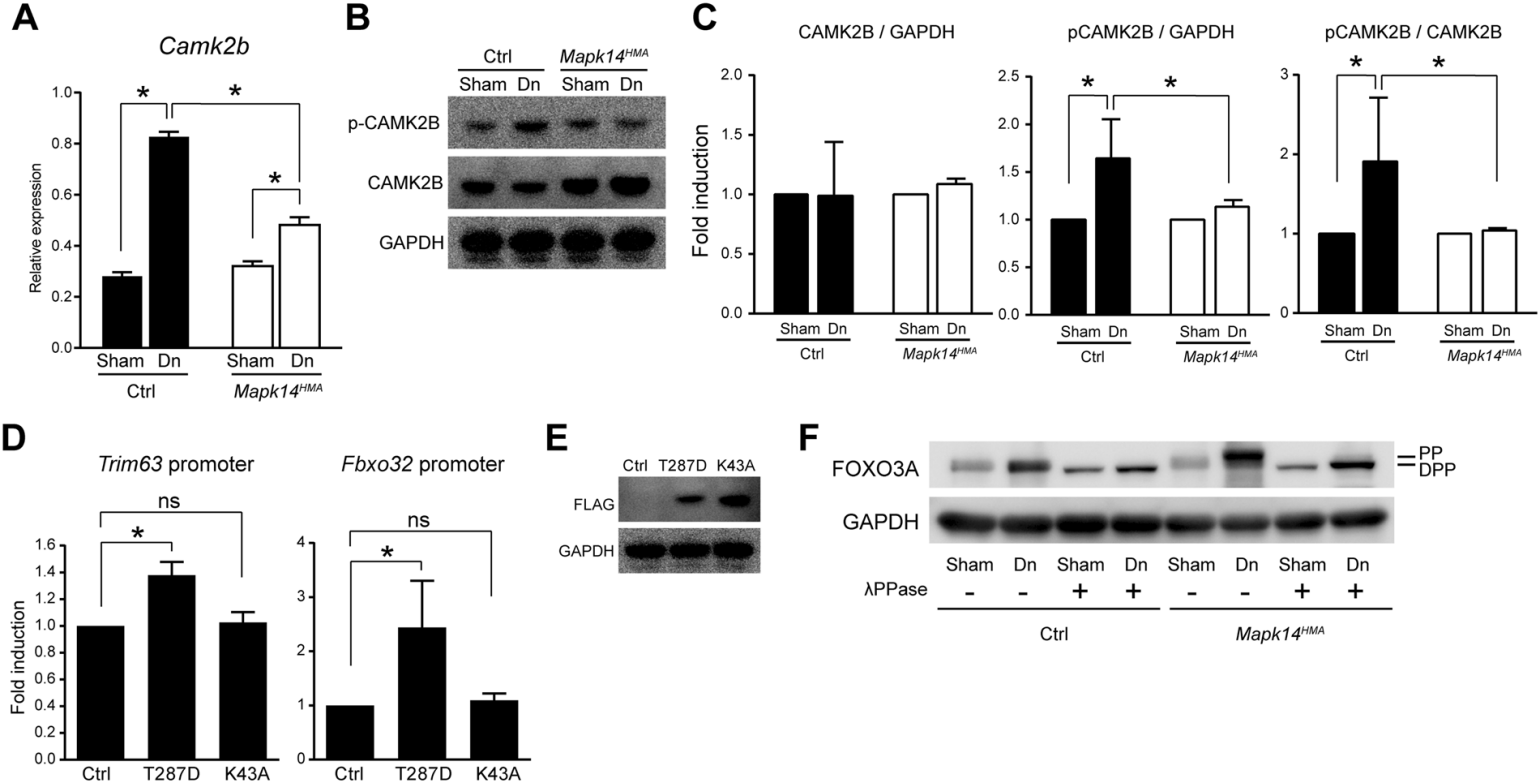
CAMK2B functions downstream of p38α MAPK to promote the transcriptional activity of *Trim63* and *Fbxo32*. **(A**) Relative expression of *Camk2b* transcripts in the TA muscle 4 d after treatment. n = 6 (Ctrl) and 7 (*Mapk14*^*HMA*^) mice. (**B**) Expression of phosphorylated and total CAMK2B in the TA muscle 4 d after treatment. Representative results of four independent experiments are shown. **(C**) Quantification of the band intensity ratios (total CAMK2B: GAPDH, phosphorylated CAMK2B: GAPDH, and phosphorylated CAMK2B: total CAMK2B). n = 4 mice per genotype. **(D**) Luciferase reporter assay using *Trim63* or *Fbox 32* reporter constructs and expression vectors harboring a constitutively active (T287D) or inactive (K43A) CAMK2B mutant. (−), Empty vector. Fold induction of luciferase activity per construct is presented. E, expression of exogenously introduced CAMK2B mutants in C2C12 cells. Representative results of 2 independent experiments are shown. F, FOXO3A expression in TA muscle collected from Ctrl and *Mapk14*^*HMA*^ mice 4 d after treatment. Tissue lysates were treated with or without λPPase. PP and DPP indicate phosphorylated- and dephosphorylated FOXO3A, respectively. n = 5 mice per genotype. Sham, sham surgery; Dn, denervation. ns, not significant, *p < 0.05.

To further understand the mechanism underlying the role of p38α MAPK in muscle atrophy, we performed a comparative gene expression analysis using muscle tissues from the Ctrl and *Mapk14*^*HMA*^ mice. The preliminary results identified several candidate genes that are differentially regulated in the *Mapk14*^*HMA*^ mice compared with their expression in the Ctrl mice after denervation (data not shown). Among the genes examined, the transcripts for *Camk2b*, which encodes calcium/calmodulin-dependent protein kinase II beta, were highly induced in the Ctrl mice after denervation. Although no significant difference was observed in the expression levels of *Camk2b* in the sham-operated limbs between the Ctrl and *Mapk14*^*HMA*^ mice, this induction after denervation was significantly decreased in the absence of p38α MAPK (Fig. 4A). Furthermore, according to a Western blot analysis of muscle tissues from the Ctrl and *Mapk14*^*HMA*^ mice, the expression of phosphorylated CAMK2B increased in the Ctrl mice but not in the *Mapk14*^*HMA*^ mice (Fig. 4B,C), indicating that CAMK2B is activated after denervation, and this activation is at least partially dependent on p38α MAPK. In contrast to the increased expression of *Camk2b* after denervation, no significant increase in the total amount of CAMK2B protein was observed, suggesting that the turnover of the CAMK2B protein is potentially enhanced after denervation.

Because the activation of CAMK2B is closely associated with the activation of p38α MAPK in denervated muscles, we investigated whether CAMK2B affects the transcriptional activity of *Trim63* and *Fbxo32*. Thus, we generated two expression vectors each bearing a construct for a constitutive active (T287D) or inactive (K43A) form of CAMK2B. These vectors were introduced into C2C12 cells with luciferase reporter vectors harboring the *Trim63* or *Fbxo32* gene promoter regions. The reporter activity of the *Trim63* promoter was significantly induced in the presence of the constitutively active CAMK2B mutant (Fig. 4D). In contrast, no increase in the reporter activity was observed in the cells transfected with the inactive CAMK2B mutant. Similar results were obtained using the reporter vector harboring the *Fbxo32* promoter (Fig. 4D). The expression of the exogenous CAMK2B mutants was confirmed by Western blotting (Fig. 4E; both mutants are FLAG-tagged to facilitate detection).

Because CAMK2B promotes the transcription of *Trim63* and *Fbxo32*, we investigated whether the FOXO transcription factors were involved in this activity. The muscle tissues were lysed and incubated with or without lambda protein phosphatase (λPPase). Because phosphorylated FOXO3A (i.e., the inactive form) migrates more slowly in the gel than dephosphorylated FOXO3A (i.e., the active form), the activity state of FOXO3A can be evaluated by performing a gel mobility shift assay. As shown in Fig. 4F, the expression of dephosphorylated FOXO3A tended to be higher in the denervated TA muscles than that in the sham-operated TA muscles in the Ctrl mice (no gel mobility shift occurred between the specimens treated with or without λPPase), but it did not reach statistical significance (data not shown). The increase in the amount of FOXO3A was also observed in the *Mapk14*^*HMA*^ mice; however, most of the protein was phosphorylated (a gel mobility shift occurred between the specimens treated with or without λPPase), indicating that the transcriptional activity of FOXO3A is reduced in the absence of p38α MAPK. Altogether, CAMK2B functions downstream of p38α MAPK to positively regulate the expression of MuRF1 and Atrogin1, and this transcriptional activity is at least partially dependent on the FOXO transcription factors.

### Pharmacological inhibition of CAMK2B attenuates denervation-induced muscle atrophy *in vivo*

**Figure 5:**
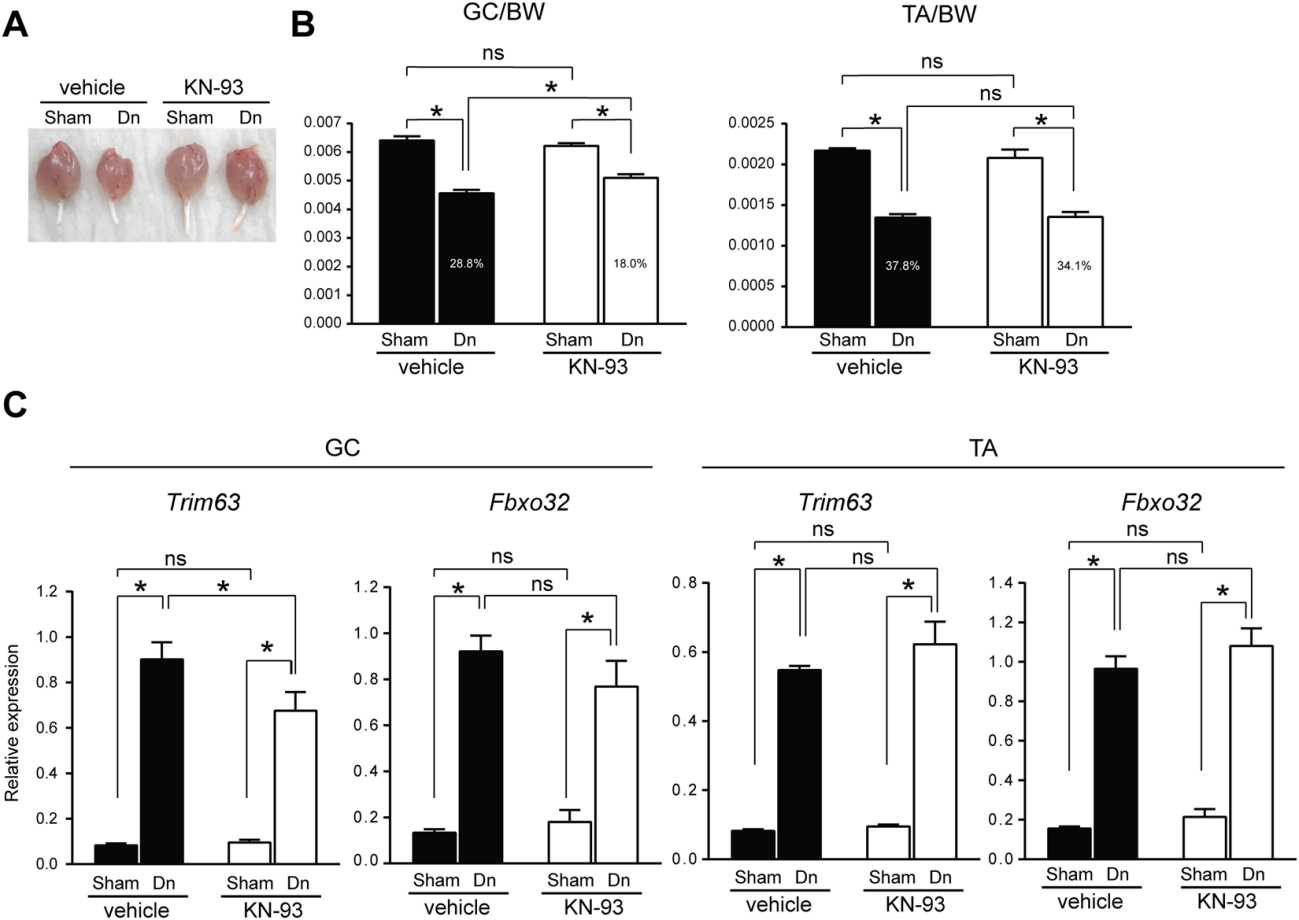
Intramuscular injection of KN-93 phosphate ameliorates denervation-induced muscle atrophy. **(A**) Gross appearance of GC muscles collected from wild-type mice 7 d after the sham surgery or denervation. Mice were treated either with vehicle or KN-93 phosphate as described in the Materials and Methods. **(B**) The GC and TA muscle to BW ratio 7 d after surgery. Numbers in the columns indicate the difference (%) between the sham-operated (set to 100%) and denervated specimens. **(C**) Relative expression of *Trim63* and *Fbxo32* transcripts in the GC and TA muscles 4 d after surgery. KN-93 phosphate was injected only into the GC muscle and not into the TA muscle. Therefore, the TA muscle specimens served as internal controls in the experiment. n = 6 mice per vehicle- and KN-93-treated group. Sham, sham surgery; Dn, denervation. ns, not significant, *p < 0.05.

The involvement of CAMK2B in the regulation of the transcriptional activity of *Trim63* and *Fbxo32* suggests that CAMK2B is a potential molecular target against muscle atrophy. To test this hypothesis, we used KN-93 phosphate, which is a potent small molecular CaMKII inhibitor, and examined whether the intramuscular administration of KN-93 phosphate attenuates denervation-induced muscle atrophy *in vivo*. Mice were treated with a daily intramuscular injection of KN-93 phosphate or vehicle (water) into the GC muscle. The contralateral limbs were sham operated and served as controls. As shown in Fig. 5A,B, the treatment with KN-93 phosphate significantly suppressed denervation-induced muscle atrophy in the GC muscle. However, no difference was observed in the weight of the TA muscle between the vehicle- and KN-93 phosphate-treated groups, indicating that the intramuscular injection of KN-93 phosphate into the GC muscle did not have systemic effects in the present model. Consistently, the denervation-induced expression of *Trim63* transcripts in the GC muscle was significantly suppressed by the treatment with KN-93 phosphate (Fig. 5C). The *Fbxo32* transcripts in the GC muscle exhibited a similar trend but did not reach statistical significance. In contrast, no marked difference was observed in the expression of the transcripts of either of the genes in the TA muscle between the vehicle- and KN-93 phosphate-treated groups (the treatments were injected into the GC muscle but not into the TA muscle). These observations support the hypothesis that the denervation-induced CAMK2B activity promotes muscle loss by inducing the expression of MuRF1.

## Discussion

In the present study, we demonstrated that p38α MAPK is activated in denervated muscles and that the muscle-specific abrogation of p38α MAPK activity yields resistance to denervation-induced muscle atrophy in mice most likely due to the suppressed expression of MuRF1 and Atrogin1 in skeletal muscle. We also identified CAMK2B as a potential downstream mediator of p38α MAPK and demonstrated that the pharmacological inhibition of CAMK2B in muscle ameliorates denervation-induced muscle loss.

Except for the suppressed muscle atrophy, no gross defects were observed in the *Mapk14*^*HMA*^ mice or the expression levels of the transcripts of MuRF1 and Atrogin1 under the unchallenged conditions, indicating that p38α MAPK is not essential for developmental or post-natal growth in the skeletal system. However, the BW of the *Mapk14*^*HMA*^ mice was lower than that of the Ctrl mice (Fig. 2C, on average approx. 9.5%). Because no differences in the ratio of muscle weight to BW (Fig. 2D) or histology (Fig. 2E) of muscle tissue were observed between the *Mapk14*^*HMA*^ and Ctrl mice, the decrease in the BW is likely not caused by defects in muscle development. However, we cannot fully exclude the possibility that p38α MAPK in skeletal muscle promotes systemic body growth potentially by producing growth factors or that the lack of p38α MAPK in cardiac muscle has a negative impact on growth.

CaMKII activity has been previously shown to increase during exercise in humans^29,30^, and activated CaMKII induces signaling pathways involved in mitochondrial biogenesis and exhibits metabolic effects in skeletal muscle^31,32^. Therefore, increased CaMKII activity is associated with muscle hypertrophy. However, in contrast, according to our data, CAMK2B functions downstream of p38α MAPK and transcriptionally promotes the expression of MuRF1 and Atrogin1. CAMK2B is activated after denervation in mice, and this activation is significantly suppressed in *Mapk14*^*HMA*^ mice (Fig. 4B,C). We initially assumed that the activation of CAMK2B was due to a compensatory mechanism to suppress overt muscle atrophy; however, the forced expression of an expression vector harboring a constitutively active form of CAMK2B induces the transcriptional activity of both *Trim63* and *Fbxo32* genes (Fig. 4D), indicating that CAMK2B functions as a catabolic regulator in skeletal muscle. Consistently, muscle atrophy and the MuRF1 transcripts were significantly suppressed by the intramuscular injection of KN-93 phosphate (please note that the transcripts for Atrogin1 showed a similar trend but the difference was not statistically significant (Fig. 5C)).

Although our data demonstrate that increased CAMK2B activity is related to muscle atrophy, we were unable to determine the mechanism by which p38α MAPK activates CAMK2B or the mechanism by which CAMK2B promotes the transcription of the *Trim63* and *Fbxo32* genes. The dephosphorylation of FOXO3A (i.e., the activation of FOXO3A) after denervation is markedly suppressed in the *Mapk14*^*HMA*^ mice, indicating that CAMK2B regulates the transcription of *Trim63* and *Fbxo32* in a FOXO transcription factor-dependent manner. However, because the FOXO transcription factors cannot be the direct targets of CAMK2B, yet-to-be identified factors likely mediate CAMK2B signaling to FOXO3A. Notably, p38α MAPK is not involved in the regulation of FOXO3A expression as evidenced by the highly induced expression levels of FOXO3A in the *Mapk14*^*HMA*^ mice (Fig. 4F).

Several limitations of the present study should be noted. First, we exclusively used a denervation model to induce muscle atrophy. Therefore, whether p38α MAPK is also involved in the development of age-related muscle loss (sarcopenia) is unclear. Second, although we were able to identify CAMK2B as a potential target of p38α MAPK in mediating muscle atrophy, unidentified factors likely exist in this putative signaling pathway as discussed above. Third, because both p38α MAPK and CAMK2B are ubiquitously expressed *in vivo*, the potential clinical application of the present findings may be limited unless a more skeletal muscle-specific target(s) is identified in this pathway.

In conclusion, the present study establishes p38α MAPK as a positive regulator of muscle atrophy and identifies an unexpected role played by CAMK2B in mediating p38α MAPK signaling in skeletal muscle. Because p38α MAPK is activated under various pathological conditions, including cachexia, sepsis, and inflammatory diseases, targeting the p38 MAPK-mediated pathway has potential in alleviating the accompanying muscle loss in these disorders. Therefore, we believe that muscle loss mediated by p38α MAPK is worthy of further investigation.

## Methods

### Mice

The generation of the *Mapk14*^*flox/flox*^ mice was performed as previously described^27^. The *Mapk14*^*flox/flox*^ mice were crossed with B6.Cg-Tg (ACTA1-cre) 79Jme/J transgenic mice in which the expression of cre recombinase is regulated under the control of a human alpha-skeletal actin promoter^28^ to specifically abrogate the *Mapk14* allele from skeletal muscle (hereafter referred to as *Mapk14*^*HMA*^ mice). Male C57BL/6 J mice were purchased from CLEA Japan (Tokyo, Japan) or Japan SLC (Shizuoka, Japan). All animal experiments were approved by the Animal Care and Use Committee of Keio University School of Medicine (approval number: 11022) and Sato Pharmaceutical Co., Ltd. All experiments were performed in accordance with relevant guidelines and regulations.

### Reagents and antibodies

We obtained KN-93 phosphate from AdooQ Bioscience (Irvin, CA). The following antibodies were used in the present study: anti-CaMKII beta (1:1000, ab107163, Abcam, Cambridge, UK), anti-ERK1/2 (1:1000, #9102, Cell Signaling Technology, Denver, MA), anti-FoxO3a (1:1000; 75D8, #2497, Cell Signaling Technology), anti-Flag (1:1000, F1804, Sigma-Aldrich, Saint Louis, MO), anti-GAPDH (1:5000; G9545, Sigma-Aldrich), anti-JNK (1:1000, #9252, Cell Signaling Technology), anti-phospho-CaMKII (Thr286) (1:1000, D21E4, #12716, Cell Signaling Technology), anti-phospho-ERK (Thr202/Tyr204) (1:1000, #9101, Cell Signaling Technology), anti-p38 (1:1000, #9212, Cell Signaling Technology), anti-phospho-JNK (Thr183/Tyr185) (1:1000, 81E11, #4668, Cell Signaling Technology), anti-phospho-p38 (1:1000, #9211, Cell Signaling Technology), anti-Mouse IgG (horseradish peroxidase-conjugated) (1:5000, 715-035-150, Jackson Immuno Laboratories, West Grove, PA), and anti-Rabbit IgG (horseradish peroxidase-conjugated) (1:5000, 111-035-144, Jackson Immuno Laboratories).

### Muscle atrophy model

Seven-week-old male mice were used in the present study. The surgical procedures were performed under general anesthesia with Isoflurane. Denervation was performed in the right hindlimb, and a sham operation was performed in the left hindlimb. The sciatic nerve was exposed through a skin incision. An approximately 3 mm long segment of the sciatic nerve was resected. The skin incision was closed using 4-0 nylon suture. For the sham operation, identical surgical procedures were performed, except for the nerve resection. The inhibition of CaMKII was achieved by a daily intramuscular injection of KN-93 phosphate (5 mg/ml, dissolved in water, 50 μl/mouse) into the gastrocnemius (GC) muscle after the intervention. Because the expression of the transcripts for MuRF1 and Atrogin1 peaks at around 3–4 d after denervation and decreases thereafter (data not shown), mice used for quantitative PCR analysis were euthanized 4 d after the intervention. On the other hand, because the muscle atrophy becomes noticeable at around 6–7 d after denervation, mice used for muscle weight analysis were euthanized on day 7.

### RNA isolation and microarray analysis

The resected skeletal muscles were lysed using Sepasol-RNA I Super G (Nacalai Tesque, Kyoto, Japan) or ISOGEN II (NIPPON GENE, Tokyo, Japan) and a bead crusher (μT-12, Taitec, Saitama, Japan or Retsch MM300, QIAGEN, Hilden, DE). The RNA was extracted according to the manufacturer’s instructions. A microarray analysis was performed using SurePrint G3 Mouse GE Microarray 8x60 K (Agilent, Saint Clara, CA).

### Quantitative PCR

Reverse-transcription was performed using ReverTra Ace qPCR Master Mix (Toyobo, Osaka, Japan). Quantitative PCR was performed using SYBR Premix Ex Taq II (Takara, Shiga, Japan) on a 7500 Real-Time PCR System or StepOnePlus Real-time PCR System (Applied Biosystems, Foster City, CA). The gene expression was normalized to the gene expression level of *Gapdh*. The quantitative PCR was performed in duplicate per sample. The nucleotide sequences of the primers used in this study are as follows: *Gapdh*, 5′-TCAACAGCAACTCCCACTCTTCC-3′ and 5′-ACCCTGTTGCTGTAGCCGTATTC-3′; *Trim63*, 5′-TACGTTGGTGCGAAATGAAA-3′ and 5′-AATCGCCAGTCACACAATGA-3′; *Fbxo32*, 5′-GTTTTCAGCAGGCCAAGAAG-3′ and 5′-TTGCCAGAGAACACGCTATG-3′; and *Camk2b*, 5′-GCACGTCATTGGCGAGGAT-3′ and 5′-ACGGGTCTCTTCGGACTGG-3′.

### Western blotting

The tissues were lysed in lysis buffer (1% Triton X-100 in PBS) supplemented with a protease inhibitor cocktail and phosphatase inhibitor cocktail (Roche, DE). The lysed samples were resolved by SDS-PAGE and transferred to a PVDF membrane. The membranes were incubated with Blocking One (Nacalai Tesque) for 1 h at room temperature and the primary antibodies overnight at 4 °C. After several washes, the membranes were incubated with horseradish peroxidase-conjugated secondary antibodies for 1 h at room temperature. The bound antibodies were detected using ECL Prime Western Blotting Detection Reagent (GE Healthcare, UK) and visualized using ImageQuant LAS 4000mini (GE Healthcare) or Image Station 4000 R (Eastman Kodak, Rochester, NY). Please note that the anti-p38 and anti-phospho-p38 antibodies used in the present study bind to all the isoforms of p38 MAPKs. To address this issue, the positive staining for p38α MAPK and phospho-p38α MAPK was confirmed by using the tissues collected from *Mapk14*^*HMA*^ mice as negative control (Fig. 2A and data not shown). In a similar vein, the antibody used to detect phosphorylated-CAMK2B is not specific for this subtype. However, because the antibody used to detect CAMK2B was specific for this subtype, we were able to identify the bands that represent phosphorylated-CAMK2B in the present study (Fig. 4B).

### Cell culture

C2C12 myoblast-like cells were obtained from RIKEN BioResource Center (Tsukuba, Japan). The cells were maintained in Dulbecco’s modified Eagle’s medium (DMEM, Wako, Osaka, Japan or Thermo Fisher Scientific, Waltham, MA) supplemented with 20% heat-inactivated FBS (HyClone, GE Healthcare), 50–100 U/mL Penicillin and 50–100 μg/mL Streptomycin (Thermo Fisher Scientific or Nacalai Tesque) in a humidified incubator at 37 °C and 5% CO_2_.

### Plasmids

The coding region of the *Camk2b* transcript was amplified by RT-PCR using cDNA obtained from the TA muscle as a template. The PCR product was cloned into the pCMV-Tag2B vector using the *Bam*HI/*Xho*I cloning sites. The constitutively active (T287D) and inactive (K43A) forms of CAMK2B were generated using a KOD-Plus-Mutagenesis Kit (Toyobo, Osaka, Japan). The promoter regions of *Trim63* (−496/+136) and *Fbxo32* (−1908/+281) were amplified by PCR using C57BL/6 J genomic DNA as a template and cloned into the pGL4 luciferase promoter vector (Promega, Madison, WI) using the *KpnI*/*Hind*III cloning sites and the *Kpn*I/*Xho*I cloning sites, respectively. The pRL-SV40 vector, which served as a transfection efficacy control, was purchased from Promega.

### Luciferase reporter assay

The C2C12 cells were seeded at the density of 4 × 10^4^ on 48-well cell culture plates and simultaneously transfected with the *Camk2b* expression vector (100 ng), luciferase reporter vector (100 ng), and pRL-SV40 vector (1 ng) using FuGENE HD Transfection Reagent (Promega) and incubated for 48 h. The luciferase activity was detected using the Dual-Luciferase Reporter Assay System (Promega). The transcriptional activity was deduced from the ratio of firefly to *Renilla* luciferase activity.

### Statistical analysis

GraphPad Prism software (Version 5.03, La Jolla, CA) was used for the statistical analyses. Student’s *t*-test (Figs 1A,B and 2C,D), one sample *t*-test (Fig. 1D), Tukey’s post hoc test (Figs 3A,B, 4A,C and 5B,C), and Dunnett’s multiple comparisons test (Fig. 4D) were performed. p < 0.05 was considered statistically significant. The data are presented as the means ± standard errors, except for in Fig. 4D, in which the data are presented as the means ± standard deviations.

### Data availability

Primary data used in figures are available upon request.

## References

[1] Narici MV, Maffulli N (2010). Sarcopenia: characteristics, mechanisms and functional significance. British Medical Bulletin.

[2] Janssen I, Heymsfield SB, Wang ZM, Ross R (2000). Skeletal muscle mass and distribution in 468 men and women aged 18-88 yr. Journal of Applied Physiology.

[3] Barillaro C, Liperoti R, Martone AM, Onder G, Landi F (2013). The new metabolic treatments for sarcopenia. Aging Clinical and Experimental Research.

[4] Waters DL, Baumgartner RN, Garry PJ, Vellas B (2010). Advantages of dietary, exercise-related, and therapeutic interventions to prevent and treat sarcopenia in adult patients: an update. Clinical Interventions in Aging.

[5] Glass DJ (2003). Signalling pathways that mediate skeletal muscle hypertrophy and atrophy. Nature Cell Biology.

[6] Glass DJ (2010). Signaling pathways perturbing muscle mass. Current Opinion in Clinical Nutrition and Metabolic Care.

[7] Bodine SC (2001). Akt/mTOR pathway is a crucial regulator of skeletal muscle hypertrophy and can prevent muscle atrophy *in vivo*. Nature Cell Biology.

[8] Braun T, Gautel M (2011). Transcriptional mechanisms regulating skeletal muscle differentiation, growth and homeostasis. Nature Reviews: Molecular Cell Biology.

[9] Sandri M (2004). Foxo transcription factors induce the atrophy-related ubiquitin ligase atrogin-1 and cause skeletal muscle atrophy. Cell.

[10] Bodine SC (2001). Identification of ubiquitin ligases required for skeletal muscle atrophy. Science.

[11] Segales J, Perdiguero E, Munoz-Canoves P (2016). Regulation of Muscle Stem Cell Functions: A Focus on the p38 MAPK Signaling Pathway. Front Cell Dev Biol.

[12] Cuenda A, Rousseau S (2007). p38 MAP-kinases pathway regulation, function and role in human diseases. Biochimica et Biophysica Acta.

[13] Zetser A, Gredinger E, Bengal E (1999). p38 mitogen-activated protein kinase pathway promotes skeletal muscle differentiation. Participation of the Mef2c transcription factor. Journal of Biological Chemistry.

[14] Wu Z (2000). p38 and extracellular signal-regulated kinases regulate the myogenic program at multiple steps. Molecular and Cellular Biology.

[15] Xu Q (2002). p38 Mitogen-activated protein kinase-, calcium-calmodulin-dependent protein kinase-, and calcineurin-mediated signaling pathways transcriptionally regulate myogenin expression. Molecular Biology of the Cell.

[16] Brien P, Pugazhendhi D, Woodhouse S, Oxley D, Pell JM (2013). p38alpha MAPK regulates adult muscle stem cell fate by restricting progenitor proliferation during postnatal growth and repair. Stem Cells.

[17] Bernet JD (2014). p38 MAPK signaling underlies a cell-autonomous loss of stem cell self-renewal in skeletal muscle of aged mice. Nature Medicine.

[18] Cosgrove BD (2014). Rejuvenation of the muscle stem cell population restores strength to injured aged muscles. Nature Medicine.

[19] Glass DJ (2005). Skeletal muscle hypertrophy and atrophy signaling pathways. International Journal of Biochemistry and Cell Biology.

[20] Adams V (2008). Induction of MuRF1 is essential for TNF-alpha-induced loss of muscle function in mice. Journal of Molecular Biology.

[21] Li YP (2005). TNF-alpha acts via p38 MAPK to stimulate expression of the ubiquitin ligase atrogin1/MAFbx in skeletal muscle. FASEB Journal.

[22] Derbre F (2012). Inhibition of xanthine oxidase by allopurinol prevents skeletal muscle atrophy: role of p38 MAPKinase and E3 ubiquitin ligases. PloS One.

[23] Kim J (2009). p38 MAPK Participates in Muscle-Specific RING Finger 1-Mediated Atrophy in Cast-Immobilized Rat Gastrocnemius Muscle. Korean Journal of Physiology & Pharmacology.

[24] Paul PK (2010). Targeted ablation of TRAF6 inhibits skeletal muscle wasting in mice. Journal of Cell Biology.

[25] Evertsson K, Fjallstrom AK, Norrby M, Tagerud S (2014). p38 mitogen-activated protein kinase and mitogen-activated protein kinase-activated protein kinase 2 (MK2) signaling in atrophic and hypertrophic denervated mouse skeletal muscle. Journal of Molecular Signaling.

[26] Childs TE, Spangenburg EE, Vyas DR, Booth FW (2003). Temporal alterations in protein signaling cascades during recovery from muscle atrophy. American Journal of Physiology: Cell Physiology.

[27] Nishida K (2004). p38alpha mitogen-activated protein kinase plays a critical role in cardiomyocyte survival but not in cardiac hypertrophic growth in response to pressure overload. Molecular and Cellular Biology.

[28] Azhar M (2010). Myocardial deletion of Smad4 using a novel alpha skeletal muscle actin Cre recombinase transgenic mouse causes misalignment of the cardiac outflow tract. International Journal of Biological Sciences.

[29] Rose AJ, Hargreaves M (2003). Exercise increases Ca2+-calmodulin-dependent protein kinase II activity in human skeletal muscle. Journal of Physiology.

[30] Rose AJ, Frosig C, Kiens B, Wojtaszewski JF, Richter EA (2007). Effect of endurance exercise training on Ca2+ calmodulin-dependent protein kinase II expression and signalling in skeletal muscle of humans. Journal of Physiology.

[31] Chin ER (2004). The role of calcium and calcium/calmodulin-dependent kinases in skeletal muscle plasticity and mitochondrial biogenesis. Proceedings of the Nutrition Society.

[32] Al-Shanti N, Stewart CE (2009). Ca2+/calmodulin-dependent transcriptional pathways: potential mediators of skeletal muscle growth and development. Biological Reviews of the Cambridge Philosophical Society.

